# Age-related changes in causal interactions between cortical motor regions during hand grip

**DOI:** 10.1016/j.neuroimage.2011.11.025

**Published:** 2012-02-15

**Authors:** Marie-Hélène Boudrias, Carla Sá Gonçalves, Will D. Penny, Chang-hyun Park, Holly E. Rossiter, Penelope Talelli, Nick S. Ward

**Affiliations:** a Sobell Department of Motor Neuroscience and Movement Disorders, UCL Institute of Neurology, London, UK; b Wellcome Trust Centre for NeuroImaging, UCL Institute of Neurology, London, UK

**Keywords:** Ageing, Motor system, Connectivity, fMRI, DCM

## Abstract

Brain activity during motor performance becomes more widespread and less lateralized with advancing age in response to ongoing degenerative processes. In this study, we were interested in the mechanism by which this change in the pattern of activity supports motor performance with advancing age. We used both transcranial magnetic stimulation (TMS) and functional magnetic resonance imaging (fMRI) to assess age related changes in motor system connectivity during isometric hand grip. Paired pulse TMS was used to measure the change in interhemispheric inhibition (IHI) from contralateral M1 (cM1) to ipsilateral M1 (iM1) during right hand grip. Dynamic Causal Modelling (DCM) of fMRI data was used to investigate the effect of age on causal interactions throughout the cortical motor network during right hand grip. Bayesian model selection was used to identify the causal model that best explained the data for all subjects. Firstly, we confirmed that the TMS and DCM measures both demonstrated a less inhibitory/more facilitatory influence of cM1 on iM1 during hand grip with advancing age. These values correlated with one another providing face validity for our DCM measures of connectivity. We found increasing reciprocal facilitatory influences with advancing age (i) between all ipsilateral cortical motor areas and (ii) between cortical motor areas of both hemispheres and iM1. There were no differences in the performance of our task with ageing suggesting that the ipsilateral cortical motor areas, in particular iM1, play a central role in maintaining performance levels with ageing through increasingly facilitatory cortico-cortical influences.

## Highlights

► DCM of fMRI data was used to investigate the effect of ageing on the motor network. ► The facilitatory influence of contra-to ipsilateral M1 increases with age. ► This is consistent with results from independently acquired neurophysiological data. ► Connections between ipsilateral motor regions were more facilitatory with ageing. ► These enhanced connections might help to maintain performance levels with ageing.

## Introduction

Ageing is associated with deleterious effects at several levels of the motor system, including degeneration of widely distributed grey and white matter structures in the cerebral hemispheres and corpus callosum (Seidler et al., 2010; Minati et al., 2007). Older subjects also exhibit decreased excitability in descending spinal pathways with a subsequent detrimental effect on the total output of the primary motor cortex (M1) contralateral to the moving hand (Delbono, 2003; Kido et al., 2004). The consequence of these changes appears to be that task-related brain activity during motor performance becomes more widespread and less lateralized with advancing age (Hutchinson et al., 2002; Kim et al., 2010; Mattay et al., 2002; Naccarato et al., 2006; Ward and Frackowiak, 2003; Ward et al., 2008). These studies allow us to see age-related changes in regional brain activity but they do not tell us about the causal interactions between brain regions that would really help in understanding the mechanisms of age-related brain reorganisation.

Studies with transcranial magnetic stimulation (TMS) can examine the causal influence of cortical brain regions on M1 contralateral (cM1) to the moving hand. For example, during hand grip cM1 inhibits ipsilateral M1 (iM1) via transcallosal connection; an effect which diminishes with advancing age (Talelli et al., 2008b). This loss of interhemispheric inhibition (IHI) is associated with greater task related brain activity in iM1 (Talelli et al., 2008a; Ward et al., 2008). TMS however does not allow the examination of causal interactions between non-primary brain regions.

Dynamic Causal Modelling (DCM) is a method of analysing data from a dynamic system such as the brain to determine which of several pre-specified causal anatomical models best fits the data (Friston et al., 2003). Bayesian inversion of the models given the empirical data allows us to measure causal interactions between brain regions through the model parameters. Previous work has applied DCM to functional magnetic resonance imaging (fMRI) data to demonstrate the effect of changing motor task (uni- or bimanual) on the causal interactions between cortical brain regions (Grefkes et al., 2008).

Here we applied DCM to fMRI data acquired during right hand grips to examine age-related changes in causal interactions within the cortical motor network. We were specifically interested in interactions between the main contributors to the corticospinal tract, in particular M1, supplementary motor area (SMA) and both dorsal and ventral premotor cortices (PMd and PMv). We expected that during right hand grip the influence of contralateral (left) M1 on ipsilateral (right) M1, assessed using DCM, would become less inhibitory/more facilitatory with age as previously shown with paired pulse TMS (Talelli et al., 2008b). Furthermore, we proposed that the increasingly bilateral task-related brain activity seen with ageing would be explained by the increasing influence of all ipsilateral brain regions, and particularly M1, on motor network activity. This approach is likely to provide a more complete mechanistic understanding of the effect of age on the causal interactions between cortical brain regions during motor performance.

## Material and methods

Twenty seven healthy volunteers participated in this study (age range = 19 to 77 years; mean age = 41.8 years ± 19.1 years Standard Deviation; 20 males). All subjects were right-handed according to the Edinburgh handedness scale (Oldfield, 1971). They reported no history of neurological illness, psychiatric history, vascular disease or hypertension, and were not taking regular medication. Full written consent was obtained from all subjects in accordance with the Declaration of Helsinki. The study was approved by the Joint Ethics Committee of the Institute of Neurology, UCL and National Hospital for Neurology and Neurosurgery, UCL Hospitals NHS Foundation Trust, London.

This dataset has previously been analysed using a general linear model (GLM) approach and reported elsewhere (Talelli et al., 2008a; Ward et al., 2008). Re-analysis of the original data using DCM affords an opportunity to make novel inferences about the effects of ageing on distributed brain systems, which was not previously possible. TMS measurements were available on a subset of 19 subjects who underwent fMRI.

### FMRI scanning

#### Experimental paradigm

All subjects underwent a single scanning session. During scanning, all subjects performed a series of visually cued dynamic isometric hand grips with the dominant right hand using a MRI-compatible manipulandum as previously described by Talelli et al. (2008a). The manipulandum consisted of two force transducers situated between two moulded plastic bars (Honeywell FSG15N1A, Honeywell, NJ, USA), which when compressed generated a differential voltage signal, linearly proportional to force exerted. Prior to scanning, each subject gripped the manipulandum using maximum force to generate a maximum voluntary contraction (MVC) for each hand. A single scanning session comprised 100 visually cued hand grips (20 each at 15%, 25%, 35%, 45% and 55% of MVC) interspersed with 60 null events (in order to introduce jitter) in a randomised and counterbalanced order (stimulus-onset asynchrony (SOA) = 3.77 s). Continuous visual feedback about the force exerted was provided. To look for bilateral movements during scanning, subjects held identical hand grip manipulanda in both hands whilst carrying out the task unimanually. The dynamic change in recorded signal was projected in real time onto a screen, which allowed detection of mirror movements.

#### Data acquisition

A 3T Siemens ALLEGRA system (Siemens, Erlangen, Germany) was used to acquire both T_1_-weighted anatomical images and functional T_2_*-weighted MRI transverse echo-planar images (EPI) (64 × 64 3 × 3 mm pixels, TE = 30 ms) with BOLD contrast. Each EPI comprised forty eight 2 mm thick contiguous axial slices taken every 3 mm, positioned to cover the whole cerebrum, with an effective repetition time (TR) of 3.12 s per volume. In total, 202 volumes were acquired during each scanning session. The first six volumes were discarded to allow for T_1_ equilibration effects.

#### Data preprocessing

All data were analysed using Statistical Parametric Mapping (SPM8, Wellcome Department of Imaging Neuroscience, UK (http://www.fil.ion.ucl.ac.uk/spm)), implemented in Matlab 2008b (The Mathworks Inc., USA). For each subject, the functional images were, in this order: realigned to the first image and unwarped to account for movement artefacts (Andersson et al., 2001); co-registered to the subject's structural image; normalized to the Montreal Neurological Institute (MNI) reference brain and smoothed with an isotropic 4 mm full-width half-maximum Gaussian kernel to account for intersubject anatomical differences and allow valid statistical inference according to Gaussian random field theory (Friston et al., 1995b). This narrow smoothing kernel was used in order to facilitate identification of separate peaks of activity in neighbouring cortical regions (e.g. M1 and PMd). The time series in each voxel were high pass filtered at 1/128 Hz to remove low frequency confounds and scaled to a grand mean of 100 over voxels and scans within each session.

#### Statistical analysis

For single subject analysis, we defined two covariates. Firstly, all hand grips were defined as a single event type and modelled as delta functions (grip covariate). A second covariate (force covariate) comprised a delta function scaled by the measured peak force exerted for each hand grip as described previously in Talelli et al. (2008a). Both covariates were convolved with a canonical synthetic haemodynamic response function and used in a general linear model (Friston et al., 1998) together with a single covariate representing the mean (constant) term over scans. Thus for each subject, voxel-wise parameter estimates for each covariate resulting from the least mean squares fit of the model to the data were calculated. The statistical parametric maps (SPMs) of the t statistic resulting from linear contrasts of each covariate (Friston et al., 1995a) were generated and stored as separate images for each subject.

### Dynamic causal modelling

DCM allows the modelling of interactions between neuronal populations by constructing a realistic model of interacting cortical areas. The model parameters in the *A* matrix describe the intrinsic connectivity among brain ROIs during the course of the experiment. The model parameters of the *B* matrix represent the change in intrinsic connectivity due to an experimental variable, in our case alteration of grip force. The model parameters in the *C* matrix represent the strength of influence of the experimental input, in our case, initiation of right hand grip The *C* matrix specifies which inputs can drive changes in which regions (e.g. *C*^*ij*^ specifies how activity in region *i* changes in response to input *j*). In other words, it represents the direct influence of initiating the hand grip on regional activity. In the standard GLM based analysis hand grips were modelled as delta functions with onset at the point of grip initiation. They were modelled as a single event type and for this reason the visual cortex was not included in our DCM models. Previous work with this paradigm did not show any age-related delay in haemodynamic response during hand grip in any of these regions (Ward et al., 2008). Non-zeros entries in the matrices [*A*,*B*,*C*] specify our assumptions about model structure. They define the functional architecture and interactions among brain regions at a neuronal level. This model is supplemented with a forward model of how neuronal activity is transformed into the measured fMRI response. DCM models the instantaneous change of the neural state vector *x*, the neurodynamics being described by the following differential equation (Friston et al., 2003):

$$\frac{dx}{dt}=\left(A+\sum_{j=1}^{m}u_{t}\left(j\right)B^{j}\right)x+C$$

where *u* denotes the experimental inputs. For our DCMs, the inputs are externally cued hand grips (driving input) and variation in grip force (modulatory input). The parameters are estimated such that the modelled BOLD signals are maximally similar to the experimentally measured BOLD signals. This enables the effective connectivity to be estimated from observed data. Estimated connection parameters describe the direction and strength of influence between the brain regions. In addition this method can provide an inference as to which model is most likely given the data (Penny et al., 2004, 2010).

Here we used DCM8 to infer and estimate the causal interactions among cortical motor regions during hand grip from measured fMRI data. We also describe how these interactions change under the influence of an external perturbation, namely event related hand grip with parametric modulation of the force produced. The aim was to look at the changes in causal effects of regions on one another with advancing age. Coupling parameters for between region connections were derived for each subject. We then used (i) age, or (ii) a TMS measure (acquired outside the scanner on the same subjects) of the change in IHI from left to right motor cortex during hand grip, to explain between-subject variability in our DCM measures.

#### Regions of interest

Eight regions of interest (ROI) were selected for DCM analysis for each subject. These included M1, SMA, PMd and PMv in both hemispheres. This selection was based on cortical areas known in primates (i) to contain corticospinal neurons (Dum and Strick, 1991), (ii) to be cortico-cortically connected with each other (Boussaoud et al., 2005; Dum and Strick, 2005; Luppino et al., 1993; Marconi et al., 2003; Rouiller et al., 1994) and (iii) to have the largest effect on activity in hand muscles (Maier et al., 2002; Boudrias et al., 2010a, 2010b). In human these areas have been shown to be consistently activated during whole hand isometric grip (Talelli et al., 2008a; Ward et al., 2003, 2008).

ROIs were selected for each subject based on individual anatomical landmarks. An F-contrast was performed across both grip and force covariates for each subject. Peak coordinates for each of M1, SMA, PMd, and PMv were identified in each hemisphere using known anatomical landmarks as a guide (Amiez et al., 2006; Fink et al., 1997; Johansen-Berg et al., 2004; Klein et al., 2007; Picard and Strick, 1996; Tomaiuolo et al., 1999). The data comprised the first eigenvariate of the BOLD time series extracted from 4 mm diameter spheres centred on these coordinates. We used 4 mm diameter spheres in order to avoid defining overlapping ROIs from M1 and PMd in the same hemisphere or SMA in both hemispheres.

#### Model selection and family analysis

Whilst exploration of the full model space is perhaps desirable, this would comprise over 6 × 10^9^ models per subject. Our goal was to determine the best or most likely structure for each of the *A*, *B* and *C* matrices and in order to reduce the model space to be tested we used a combination of (i) a priori knowledge about the motor network under investigation together with (ii) model selection procedures for when this information was not available. Although this is presented as a sequential process (determining *A* then *B* matrices), in fact it is possible to retest assumptions made through model selection at any stage.

**Fig. 1:**
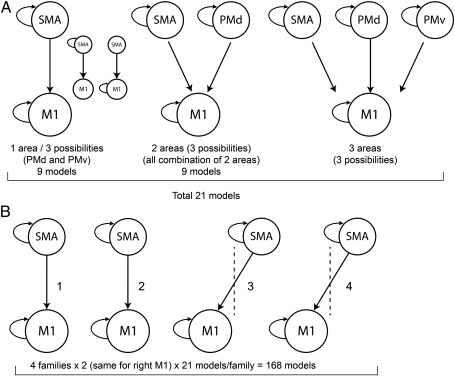
Specification of B matrix models. A) Examples of alternative B matrix models created in one typical family. In this example, alternative models testing for the modulation by grip force of effective connectivity of one, two or three secondary motor areas with M1 of the same hemisphere were created. There were three variations for each model created. One variation allowed for all the areas in the model to be self-connected (shown here). Two other models allowed for M1 or the secondary motor present to be self-connected (represented in smaller format). When more than one supplementary motor area was present in a given model and the self-connection tested, the feature was applied to all of them. B) Using the methodology described above, 4 variations of the family of models described in A were created using left M1 (families 1–4, shown here) and 4 variations using right M1 (families 5–8, not shown were). Thus, twenty one models were created in each of these 8 families. A total of 168 models distributed in 8 families were created for this analysis. Dotted line represents the midline.

Cortico-cortical connections between our selected motor ROIs are known to exist, both within (Dum and Strick, 2005; Luppino et al., 1993) and between (Boussaoud et al., 2005; Liu et al., 2002; Marconi et al., 2003) hemispheres in primates. Therefore, a priori, no restrictions were made in the intrinsic connections (*A* matrix) of our tested models. In order to test for the effect of changing grip force (*B* matrix) we created a total of 168 models divided in 8 different families for each subject (see Fig. 1). Our previous experience with this paradigm suggests that activity in M1 and SMA is modulated by changing force (Ward and Frackowiak, 2003; Talelli et al., 2008a, 2008b; Ward et al., 2008). Furthermore, we have seen that activity in the premotor areas PMd and PMv is also modulated by changing force in older subjects and after stroke (Ward et al., 2007, 2008). Based on these data, model families were created in a systematic way to explore how the connections between secondary motor areas and M1 are modulated by force and how these effects are modulated by age. As the visual cortex was not the main concern of the hypotheses being tested, it was not included in our DCM models. We reasoned that the input should therefore be in all ROIs in the left hemisphere.

Bayesian model selection (BMS) was used to determine the most likely among a set of candidate models given the observed fMRI data. In that context, the optimal model represents the best balance between model fit and model complexity (Stephan et al., 2007). Model comparison and selection rest on the model evidence, in other words, the probability of observing experimentally measured BOLD signals under a particular DCM model. Selection of the optimal model family was based on the assumption that it would be identical across subjects and that only the connection strengths would be modified with age .This ‘fixed effects’ (FFX) inference assumes that the basic physiological mechanism for the production of handgrip was unlikely to vary across subjects. This assumption is supported by fMRI studies of healthy subjects showing age-related changes in the magnitude of hand grip related brain activation, but not in the topology of the network itself (Talelli et al., 2008a, 2008b; Ward and Frackowiak, 2003; Ward et al., 2008). FFX inference was therefore used to make model inference at the group level. They are reported using Group Bayes Factors (GBF) (Stephan et al., 2007).

### Transcranial magnetic stimulation

The IHI values used in this study have been already reported and discussed in detail elsewhere (Talelli et al., 2008b). Not all subjects were tested for IHI with TMS. Here we report the IHI value of 19 subjects out of our 27 subjects who underwent fMRI. Briefly, IHI from left to right M1 during right hand squeezing was measured using paired coil TMS and was shown to positively correlate with age. IHI is expressed as the reduction in the response of the right M1 following the delivery of a suprathreshold TMS test pulse when another suprathreshold conditioning pulse is delivered to the left M1, in this case 40 ms earlier. Excitability of the right M1 expressed in terms of motor evoked potentials (MEPs) was measured from the left first dorsal interosseous (FDI) from ten single (test) and ten paired-pulse (conditioning + test) trials. IHI was defined as the conditioned/test MEP amplitude ratio. IHI was initially measured at rest (group average value of 0.575). The stimulation intensity for both the conditioning and the test stimuli was adjusted to evoke an MEP of 1–1.5 mV in the contralateral FDI muscle. For the active condition (activeIHI) the subjects were instructed to contract the right FDI to 15–20% of their MVC in response to an auditory cue preceding the conditioning stimulus by 600 ms (group average value of 0.559; smaller value means more IHI). The stimulation intensity both for the conditioning and test stimulus was the same as in the resting state, as in previous studies (Ferbert et al., 1992). The absolute values of active IHI were then expressed as a ratio of the values at rest (change IHI). Change IHI therefore reflects the change seen in the IHI targeting the right motor cortex when the right hand is active. Values < 1 reflect stronger inhibition, while values > 1 reflect less inhibition.

## Results

### Main effects of handgrip

The average force (34.6% of MVC) and duration (1918 ± 123 ms) of hand grips were calculated for all trials. The average error in achieving the target force was 2.5 ± 0.6% of MVC. There was no correlation between age and any of these performance parameters. The main effects of hand grip (see Supplementary material (*i*)) were consistent with previous studies that used the same paradigm and not described in detail here (Talelli et al., 2008a; Ward and Frackowiak, 2003; Ward et al., 2008). Task-related changes in activation were observed in a distributed network including the motor areas of both hemispheres selected in our DCM analysis: M1, PMd, PMv and SMA.

### Anatomical regions of interest

The resulting coordinates of the extracted ROIs were consistent across subjects. The variability is expressed in terms of standard deviation (SD). The average coordinates are given in Table 1 and in agreement with previous literature (Amiez et al., 2006; Fink et al., 1997; Johansen-Berg et al., 2004; Picard and Strick, 2001; Tomassini et al., 2007). Individual coordinates of ROIs can be found in Supplementary material (*ii*).

### Family and model selection

**Fig. 2:**
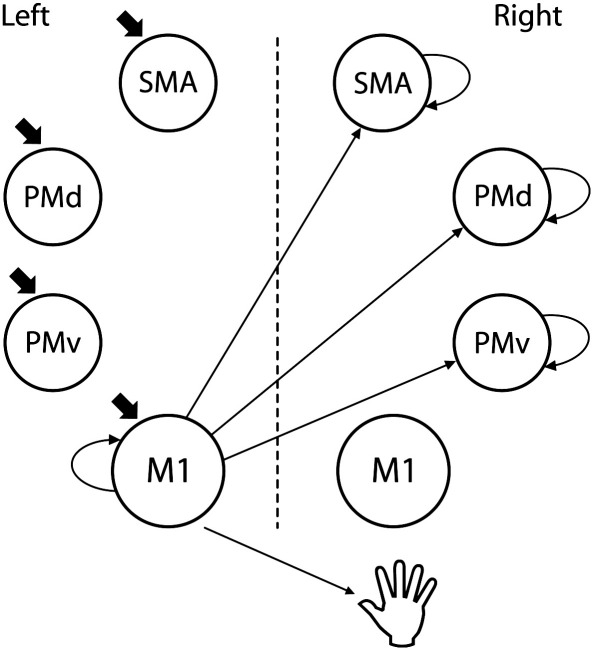
Features of the winning model from which coupling parameters were extracted in every subject for further correlation analysis. The A matrix includes a fully connected matrix (not shown here). The connections shown here are those which decrease by changing grip force (as derived from the B matrix). The site of influence of the experimental input (initiation of right hand grip, derived from C matrix) is represented by the small thick arrows.

As described above we assumed that intrinsic connections (*A* matrix) existed between each ROI and all the others. Next, we assumed that the experimental input (‘go’ cue) targeted all left sided ROIs. In order to determine the optimal *B* matrix that could best account for changes in BOLD signal when different levels of grip force were used, we created 168 competing models contained in 8 different families (Fig. 1). The winning family included models with connections between left M1 and the secondary motor areas (SMA, PMd and PMv) of the right hemisphere (Fig. 2). This family showed the greatest log Bayes factors of 1.07 + 14, far superior to the next best family with a log evidence value of 18. This corresponds to very strong evidence in favour of the winning family (family 4 of Fig. 1) (Penny et al., 2004). Bayesian model average (BMA) was performed on the models contained in the same winning family for each subject (Penny et al., 2010). In this method, the contribution of each model to the mean effect is weighted by its evidence. The coupling parameters of the mean model computed were extracted and used for the all subsequent correlation analysis described below.

### Explaining variation in coupling parameters

#### Overall average of all models

We performed BMA of the 21 models included in the winning family for all subjects. This allowed the evaluation of the overall network connectivity profile during right hand grip for all subjects. We observed that on average, all values for intrinsic connectivity were positive during grip (*A* matrix) with the exception of those targeting right M1 (Table 2). With the exception of left M1, all motor areas had an overall inhibitory influence on right M1. The connectivity values between left M1 and the other motor areas were the largest ones observed in our network (with the exception of that targeting right M1). Connectivity values between left SMA and the other motor areas (except for right M1) were the second largest ones after those originating from left M1 (between 0.05 and 0.07).

The average inputs to the motor system during the hand grip were also all positive. M1 showed the largest value (1.26) compared to the other areas (SMA = 0.1, PMd = 0.04 and PMv = 0.08). The connection strength modulated by changing force (*B* matrix, see Supplementary material (*iii*)) from left M1 to right SMA, PMd, and PMv all showed an overall decrease with increasing force (30%, 33% and 30% respectively). The self connections did not change more than 1.5% during modulation of force.

#### Age and interhemispheric inhibition

**Fig. 3:**
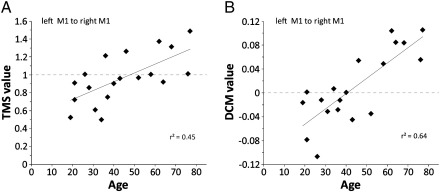
Linear correlations between age and TMS and DCM-derived values of IHI. A) TMS values reflect change in IHI from left to right M1 expressed as a ration of active to rest values. TMS values < 1 reflect stronger inhibition, whilst values > 1 reflect less inhibition. B) DCM values represent the strength and sign of the coupling between left M1 to right M1 during the performance of the same task. Negative values = inhibition, positive values = facilitation (n = 19 as not all subjects were tested for IHI with TMS).

Using TMS, Talelli et al. (2008a, 2008b) showed that the interhemispheric influence of left M1 on right M1 during right hand grip is inhibitory in younger subjects but becomes less so, and even facilitatory, with advancing age (Fig. 3A, r^2^ = 0.45, *p* < 0.05). In the same subjects, we found that the DCM-derived coupling parameters from left M1 to right M1 (derived from each subject's *A* matrix) also correlated with age (Fig. 3B, r^2^ = 0.64, *p* < 0.001). For the majority of older subjects cM1 exerted a facilitatory effect on iM1 but the effect was largely inhibitory in younger subjects. When comparing the coupling parameters of intrinsic connectivity between cM1 and iM1 during a unilateral motor task, the results for our younger subjects were broadly similar to those obtained by Grefkes et al. (2008). The respective TMS and DCM measures of the influence of left M1 on right M1 during right hand grip were also correlated (r^2^ = 0.31, *p* < 0.05).

#### Age and network connectivity

**Fig. 4:**
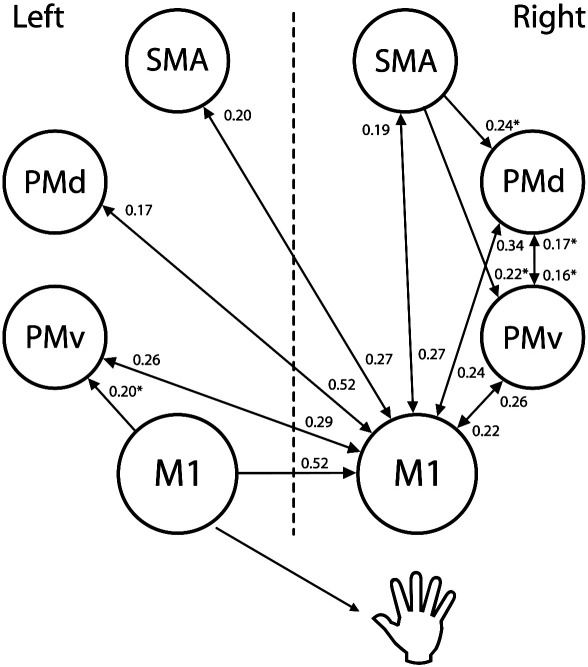
Connections for which coupling parameters during right hand grip become less inhibitory/more facilitatory with age. Values are given as r2 (all *p* < 0.05, except for right M1 to right SMA *p* < 0.0507) and are extracted from the A matrix (n = 27). Asterisk indicates DCM values of the A matrix that were all positive (with the exception of 1–3 subjects) and became more positive with age. Note that the correlation value for between left M1 and right M1 is different than the one of Fig. 3 because it includes data from a larger number of subjects.

Our current approach allowed us to investigate whether hand grip related coupling parameters between other cortical motor regions were also affected by age. Fig. 4 illustrates connections for which coupling parameters derived from each subject's *A* matrix became less inhibitory/more facilitatory with age. Most of these involve reciprocal connections between all motor areas and iM1 (right), or intrahemispheric connections in the right (ipsilateral) hemisphere. We identified two main types of relationships between the changing of intrinsic coupling and age. The first corresponded to a reduction in inhibition where values were negative at a young age but become less negative or positive at an older age. This was the pattern observed for the majority of the connections. The second type of pattern involved an increase in facilitation where the DCM values from the *A* matrix were all positive (with the exceptions of 1 to 3 subjects) and became more positive with age. Fig. 3B shows an example of type 1 pattern. Connections with type 2 patterns are indicated in Fig. 4.

#### The effect of changing force on network connectivity

**Fig. 5:**
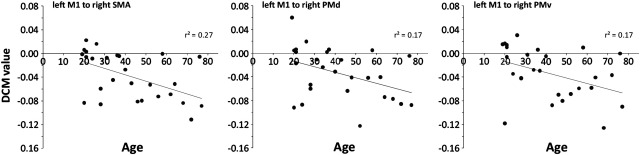
Correlation between modulation of connection strength and age during production of hand grip force. Correlations have p-value of 0.002, 0.0539 and 0.0611 respectively (B matrix, n = 27).

We also examined the *B* matrix values for the 3 connections in which connection strength was found to be modulated by changing force — left M1 to right SMA, PMd and PMv (Fig. 2). The *B* matrix describes how the measures of effective connectivity during hand grip (*A* matrix) are modulated when the grip force changes. A negative value in the *B* matrix indicates a decrease in coupling with increasing force production. We examined whether these values correlated with age. A negative correlation was found between age and the modulation of connection strength from left M1 to right SMA during gripping. The modulation of connection strength from left M1 to both right PMd and PMv during gripping also showed a trend towards correlating with age (Fig. 5). In all cases connection strength was more likely to be decreased by increasing grip force in older subjects.

## Discussion

In this study, we were interested in how the causal interactions that take place between cortical brain regions during the performance of a simple motor task were affected by advancing age. In general, previous studies suggest that older subjects have less lateralised brain activity across a range of tasks (Cabeza, 2001). In the motor domain, the normal younger pattern of decreased activity in M1 ipsilateral to the moving hand (iM1) is often reversed with ageing (Ward et al., 2008). A paired pulse TMS study suggested that this reversal is due to reduced task-related interhemispheric inhibition from cM1 to iM1 (Talelli et al., 2008b). Here, we found the same age related phenomenon using DCM of fMRI data. DCM coupling parameters have not previously been used to investigate variability in the causal influences between brain regions, but a significant correlation between the DCM and TMS derived measures of cM1 to iM1 connectivity suggests that these measures are reliable and provides face validity for our approach. This is particularly important when examining connectivity between the non-primary cortical regions in our anatomical model that cannot be assessed with paired pulse TMS.

Our results indicate that with advancing age there is a reciprocal decrease in inhibitory/increase in facilitatory influence between (i) all ipsilateral cortical motor areas and (ii) cortical motor areas of both hemispheres and iM1. In other words, cortical motor areas within the ipsilateral hemisphere, in particular M1, become increasingly involved during the performance of a simple motor task with ageing. We chose a simple motor task that could be performed equally by all subjects in order to avoid the problem of performance confounds. There was no change in task performance with ageing and so it is likely that these changes occur in response to degenerative changes in a way that maintains performance, rather than because performance is different across subjects. In other words, these changes are likely to be compensatory.

We have confirmed the increasing importance of the ipsilateral hemisphere in maintaining motor performance in older subjects (Seidler et al., 2010; Ward, 2006). Furthermore, our results suggest that the mechanism by which iM1 plays an increasingly central role is through facilitatory connections with most other non-primary cortical brain regions. The exact role of iM1 in the generation of hand movements remains controversial. Studies in primates have shown that iM1 comprises 18% of the terminations in the cervical enlargement pointing towards an anatomical mechanism for its contribution to the control of ipsilateral hand muscles (Dum and Strick, 1996). Electrophysiological studies in healthy subjects have shown evidence for an ipsilateral projection to axial and proximal stabilising muscles rather than hand muscles (Carr et al., 1994). However, repetitive TMS to M1 results in errors in both complex and simple motor tasks using the ipsilateral hand (Chen et al., 1997) suggesting that iM1 may play a role in planning and organisation of normal hand movement.

DCM is Bayesian in all aspects and is an established procedure in statistics that rests on computing the model evidence i.e. the probability of the data given the model. The model evidence quantifies the properties of a good model, it explains the data as accurately as possible and, at the same time, has minimal complexity. It also measures the generalisability of the model to all subjects, which is represented by the likelihood of the data, having taken into account the natural variability of the model parameters. In other words, it provides a prediction of the data based on the prior density of its parameter. Posterior densities obtained for each parameter of the model provide a measure about the strength of coupling between two given areas. They also provide a measure of dependency on experimental perturbation of the coupling parameters. These posterior densities are conditional on the particular model chosen. Endogenous or intrinsic couplings as characterised by values of the *A* matrix do not represent purely anatomical connections and are conditional on the task used during acquisition of the data. This implies that unless the same task is used, these values cannot be directly compared with other studies. This is particularly true when considering interhemispheric connectivity when one hand is used (in our case) or both hands used (albeit not at the same time, as in the case of the Grefkes et al., 2008 and Rehme et al., 2011). Because the model space related to our selected ROIs could not be explored in its entirety, we used combinations of a priori knowledge about the motor network and BMS approaches in our search for the model that could best account for our data (Stephan et al., 2007). In that context, BMS was used to find the optimal family from all the alternatives tested and FFX inference made as the basic physiological mechanism for the production of handgrip was unlikely to vary across subjects. We also performed a post-hoc analysis using a random effects family inference which resulted in the same winning family as for the FFX inference (with an exceedence probability for the winning family = 0.9871). Although force modulation was not the main focus of the study, we found that the principle feature of the *B* matrix that generalised best to our subjects involved the connections from left M1 to right SMA, PMd and PMv. These connections decreased on average by a third when force was increased. Correlation analysis suggested that older subjects showed the largest decrease in these connectivity values. Again, this does not imply that other connections are not modulated with age but rather describes the features that could best account for changes in BOLD signal when different levels of grip force are produced in our cohort of subjects. DCM is currently limited to models that are made of a maximum number of eight VOIs. Thus although it might be interesting to answer questions about other regions, our primary question here was to address the inter-relationship between cortical motor areas contributing to corticospinal tracts. Alternative methods for addressing larger scale networks include graph theory (Gerloff and Hallett, 2010), but that is beyond the scope of our current manuscript.

In conclusion, our results indicate that ipsilateral motor areas, in particular iM1, become increasingly functionally relevant in the ageing brain. Whilst previous work has demonstrated age-related overactivity in iM1, probably secondary to reduced interhemispheric inhibition from cM1, here we have shown that iM1 exerts an increasingly facilitatory influence over all the non-primary cortical motor areas in our model. This result points towards the importance of causal cortico-cortical connections in maintaining performance in the face of degenerative change. Our DCM derived measures of the influence of cM1 on iM1 correlated with TMS derived measures of the same process, demonstrating that the coupling parameters themselves behave in a predictable and biologically plausible way. This correlation provides face validity when examining coupling parameters between non-primary motor regions, not accessible to TMS. Our results demonstrate that changes in the causal influence of ipsilateral cortical motor areas can explain age related variability in motor system activation during hand grip. More generally, this work once again highlights an important property of the central nervous system in that the functional influences of brain regions upon others are adaptable in behaviorally relevant ways.

## Figures and Tables

**Table 1:** Average coordinates of the extracted regions of interests (ROIs) given in MNI space. The variability is expressed in terms of standard deviation (SD).

	M1_L			SMA_L			PMd_L			PMv_L		
*x*	*y*	*z*	*x*	*y*	*z*	*x*	*y*	*z*	*x*	*y*	*z*
Average	− 38	− 21	58	− 4	− 7	59	− 28	− 8	62	− 56	5	33
SD	3.8	4.6	4.5	2.8	3.3	4.9	6.8	3.7	4.2	3.5	3.1	5.1

	M1_R			SMA_R			PMd_R			PMv_R		
*x*	*y*	*z*	*x*	*y*	*z*	*x*	*y*	*z*	*x*	*y*	*z*

Average	41	− 20	60	6	− 4	60	32	− 7	61	56	9	33
SD	7.6	6.4	5.0	2.9	5.1	4.9	6.6	3.9	5.5	3.6	3.4	5.3

**Table 2:** Average effective connectivity values for all connections during grip (*A* matrix) (from (column) to (row)).

	*M1_L*	*M1_R*	*SMA_L*	*SMA_R*	*PMd_L*	*PMd_R*	*PMv_L*	*PMv_R*
*M1_L*		0.01	0.06	0.02	0.02	0.02	0.03	0.02
*M1_R*	0.00		− 0.03	− 0.02	− 0.03	− 0.02	− 0.02	− 0.03
*SMA_L*	0.16	0.01		0.02	0.02	0.01	0.03	0.01
*SMA_R*	0.13	0.01	0.06		0.04	0.02	0.05	0.02
*PMd_L*	0.15	0.00	0.06	0.02		0.02	0.04	0.02
*PMd_R*	0.11	0.01	0.07	0.04	0.05		0.05	0.03
*PMv_L*	0.14	0.01	0.05	0.02	0.03	0.01		0.01
*PMv_R*	0.11	0.00	0.07	0.04	0.06	0.04	0.06	
