# Supplementary material for: Age-related changes in causal interactions between cortical motor regions during hand grip
Source: Neuroimage. 2012 Feb 15;59(4-4):3398–405. doi: 10.1016/j.neuroimage.2011.11.025 (PMC3315004; doi:10.1016/j.neuroimage.2011.11.025)
Supplement: Supplementary file 1 — Supplementary materials. [file mmc1.doc]

**Supplementary material**

**Age-related changes in causal interactions between cortical motor regions during hand grip**

Marie-Hélène Boudrias, Carla Sá Gonçalves, Will D. Penny, Chang-hyun Park, Holly E. Rossiter, Penelope Talelli, Nick S. Ward

*(i) Main effects of hand grip*

**
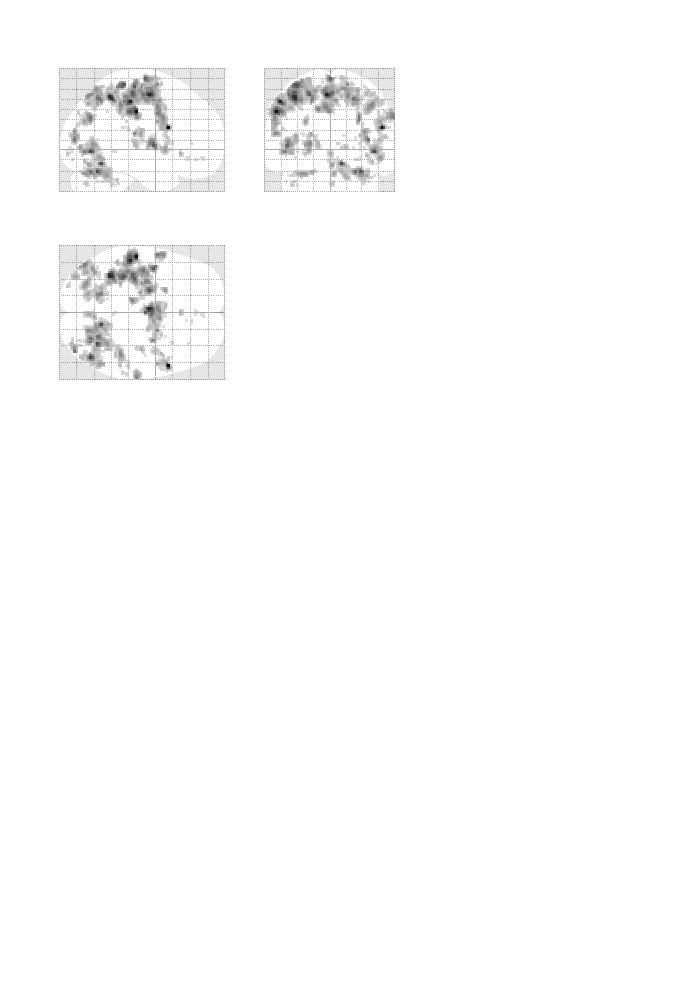
**

*SPM {z} representing the main effect of hand grip detected as canonical haemodynamic response function. Results are displayed on ‘glass brain’. The glass brain are shown from the left side (top right image), from below (top left image) from the above (bottom image). Voxels are significant at P < 0.05 (corrected).*

*(ii) Individual coordinates of ROIs*

*Coordinates are given in MNI space.*

*(iii) Average effective connectivity values modulated by changing force (B matrix) (from (column) to (row)).*

*The activity in the B matrix corresponds to the percentage change (facilitatory or inhibitory) of the activity in the A matrix per unit time.*
